# From batch to flow-through multi-frequency sonicator for PFAS degradation

**DOI:** 10.1016/j.ultsonch.2026.108008

**Published:** 2026-08-17

**Authors:** Sara Maghami, Jean Noel Uwayezu, Ivan Carabante, Örjan Johansson

**Affiliations:** a Engineering Acoustics, Division of Structural Engineering, Luleå University of Technology, Sweden; b Waste Science and Technology, Division of Geosciences and Environmental Engineering, Luleå University of Technology, Sweden

**Keywords:** Acoustic cavitation, Multi-frequency excitation, PFAS degradation, Flow-through sonicator

## Abstract

The destruction of per- and polyfluoroalkyl substances (PFAS) remains a significant environmental challenge. Acoustic cavitation has shown promising potential for PFAS degradation; however, its broader application is limited by the low treatment capacity and insufficient energy efficiency of conventional sonication systems. This study investigates the transition from batch to flow-through multi-frequency sonication. A high-frequency unit was numerically designed and acoustically optimized to enhance acoustic pressure focusing and cavitation intensity. The optimized unit was implemented in a single-frequency flow-through configuration (135 kHz, 0.2 kWh/L), achieving 66% PFOS and 55% PFOA removal. In addition, conventional batch and hybrid batch/flow-through configurations were evaluated as intermediate steps toward the development of a flow-through sonication system. The dual frequency batch system (21 and 31 kHz, 0.8 kWh/L) achieved 40% PFOS and 45% PFOA degradation, whereas the triple-frequency hybrid sonicator (21, 31, and 135 kHz, 0.8 kWh/L) achieved up to 77% PFOS and 81% PFOA removal. The progressive formation of short-chain PFAS indicated sustained chain scission during sonication. To further investigate treatment capacity and energy efficiency under larger-volume operation, a dual-frequency flowthrough reactor (21 and 38 kHz) was proposed and evaluated, achieving 74% PFOS and 36% PFOA degradation at an energy density of 0.10 kWh/L. These findings demonstrate the influence of frequency and reactor configuration on PFAS degradation mechanism and highlight the potential of multifrequency flow-through sonication to improve the energy efficiency of PFAS degradation.

## 1 Introduction

Poly- and perfluoroalkyl substances (PFAS) are persistent chemicals characterized by exceptionally strong carbon–fluorine bonds [1], [2], [3], [4]. Their resistance to degradation, environmental persistence, bioaccumulation, and potential toxicity have created an urgent need for effective treatment technologies [5], [6], [7].

Conventional separation methods, including activated carbon, ion-exchange resins, nanofiltration, and reverse osmosis, remove PFAS from water but generate concentrated waste streams requiring further treatment [8], [9], [10], [11]. Destructive approaches instead aim to cleave C–F bonds and include incineration, electrochemical oxidation, UV/persulfate treatment, and plasma processes [12], [13], [14], [15]. Acoustic cavitation is a promising alternative because it can directly degrade PFAS with limited chemical addition and secondary waste generation [16], [17], [18], [19].

Acoustic cavitation involves the growth and collapse of bubbles under ultrasonic pressure fluctuations. Bubble growth through rectified diffusion followed by inertial collapse produces localized high temperatures and pressures that promote pyrolytic and radical-mediated reactions [20], [21], [22], [23], [24]. Sonochemical performance depends on reactor geometry, acoustic power, operating frequency, and frequency configuration [25], [26].

PFAS amphiphilicity promotes accumulation at the bubble–liquid interface, where collapse can induce pyrolysis, defluorination, and chain scission. Longer-chain PFAS, including PFOS and PFOA, generally degrade more readily than shorter-chain compounds because of their greater hydrophobicity, lower solubility, and stronger interfacial adsorption [27], [28]. The formation of less-reactive short-chain products further supports sequential degradation pathways [29].

PFOS typically exhibits stronger surface activity and interfacial enrichment than PFOA [30]. PFOS degradation commonly proceeds through desulfonation and C–S bond cleavage, whereas PFOA degradation mainly involves decarboxylation [31], [32]. Radical-mediated and solvated-electron pathways may also contribute depending on the acoustic environment [26]. Thus, degradation is governed by interfacial adsorption, thermal effects, cavitation intensity, bubble dynamics, radical transport, acoustic streaming, pressure distribution, and surfactant accumulation [30], [33], [34].

Acoustic frequency strongly affects these mechanisms. Low frequencies (20–40 kHz) generate larger bubbles and stronger collapse, favoring pyrolysis, whereas mid frequencies (100–500 kHz) produce smaller bubbles with greater interfacial area and radical activity. Near 1 MHz, bubble oscillations become more stable and higher power is generally required [19], [33], [35]. Power density controls bubble population and collapse intensity, while Bjerknes forces influence bubble migration, clustering, and cavitation uniformity [36].

Multi-frequency excitation can further modify cavitation through pressure field superposition, bubble coupling, intermodulation, and harmonic interactions. These effects can broaden the active bubble population, improve pressure localization, and enhance oxidation, mass transfer, sonoluminescence, and PFOS/PFOA degradation [25], [37], [38], [39], [40]. Mid-to-high-frequency sonolysis generally follows similar degradation mechanisms, whereas low-frequency treatment is often slower and more dependent on added oxidants [19]. Harmonics and subharmonics generated by the nonlinear liquid–bubble response may also influence bubble expansion and collapse [38], [39], [41], [42], [43].

PFAS concentration also affects degradation kinetics. Sonolysis commonly follows pseudo-first-order behavior under dilute conditions and approaches zero-order behavior as the bubble interface becomes saturated [30], [31], [32]. Transition concentrations are generally 15–40 µM at mid-to-high frequencies but may be lower at 20 kHz [26], [34], [44]. Concentrations near 1 mg L^−1^ therefore generally represent dilute conditions, whereas approximately 10 mg L^−1^ may approach interfacial saturation and mass-transfer limitation. This transition also depends on frequency, power density, dissolved gas, surface tension, co-contaminants, and multi-frequency interactions [30], [45].

Despite its degradation potential, PFAS sonolysis remains largely limited to laboratory-scale batch systems because of high energy demand, low throughput, non-uniform cavitation, reactor complexity, and limited scale-up strategies [27], [46], [47]. Early experiments at 200 kHz treated 60 mL solutions.

containing 10 mg L^−1^ PFOS and PFOA at approximately 3333 W L^−1^. After 60 min, degradation reached 28% for PFOS and 63% for PFOA in air and 60% and 85%, respectively, under argon [31]. These results demonstrated feasibility but also highlighted the need to reduce specific energy consumption and increase treatment volume [47], [48], [49], [50].

Pilot-scale treatment has been demonstrated in a 91 L multi-transducer reactor operated at 500 kHz and 1 MHz with up to 12 kW input power. Treatment of approximately 30 mg L^−1^ PFAS for 13 h resulted in substantial transformation but required a nominal energy density of approximately 1.7 kWh L^−1^ [51]. This confirms the scalability of sonolysis while emphasizing the need for more efficient reactor configurations. Flow-through systems may improve treatment capacity and enable continuous operation, although comparisons with batch reactors remain limited [52], [53], [54].

Scale-up therefore requires improved acoustic-field uniformity and energy transfer. Multiple-transducer arrangements and polygonal geometries can generate overlapping cavitation regions, improve pressure distribution, and reduce localized erosion [46], [55], [56]. Efficient energy transfer also depends on coupling between structural vibration modes and acoustic modes in the confined liquid [57], [58], [59]. Appropriate modal coupling promotes constructive interference and pressure amplification [60], [61], [62].

In fluid-loaded cylindrical systems, flexural modes dominate structural– acoustic energy exchange, and coupling improves when the transverse structural impedance approaches the acoustic impedance of the liquid [61], [63], [64]. Because fluid loading shifts structural natural frequencies, both structural and fluid-domain eigenmodes must be considered during reactor design [59], [64]. Strong interaction can occur when the structural breathing mode lies below the radial acoustic eigenfrequency of the confined fluid [65], [66], [67]. Hexagonal transducer arrangements may further improve mode coupling and acoustic focusing [56], [65]. The corresponding analytical framework has been reported previously [68].

Based on these principles, the reactors investigated here were designed according to the acoustic wavelengths, structural modes, and expected pressure antinode locations of the applied frequencies [69], [70], [71]. Emphasis was placed on a high-frequency booster comprising six transducers symmetrically arranged around a central cylindrical structure. Numerical modelling in COMSOL Multiphysics was used to evaluate acoustic pressure fields, cavitation conditions, and fluid flow.

The present study investigates the transition from batch to flow-through PFAS sonolysis using acoustically optimized single- and multi-frequency reactor configurations. Single-, dual-, and multi-frequency excitation are compared in batch, flow-through, and hybrid systems to evaluate their effects on PFOS and PFOA degradation, cavitation performance, scalability, and energy efficiency. The study further examines whether lower fundamental frequencies, geometric optimization, and nonlinear multi-frequency interactions can improve pressure-field distribution and PFAS degradation in larger treatment volumes.

## 2 Materials and methods

### 2.1 Materials

The chemicals used to prepare the synthetic PFAS solutions were perfluorooctanoic acid (PFOA; 96%) and perfluorooctane sulfonate (PFOS; 96%) purchased from Sigma-Aldrich (USA). The ultrapure water was used to prepare the spiked solutions. The experimental solutions contained a mixture of PFOA and PFOS. In the SF-FT, DF-B, and TF-H experiments, each parent compound was present at an initial concentration of 1 mg L^−1^. In the DF-FT experiments, each parent compound was present at an initial concentration of 10 mg L^−1^.

### 2.2 Design methodology

**Fig. 1:**
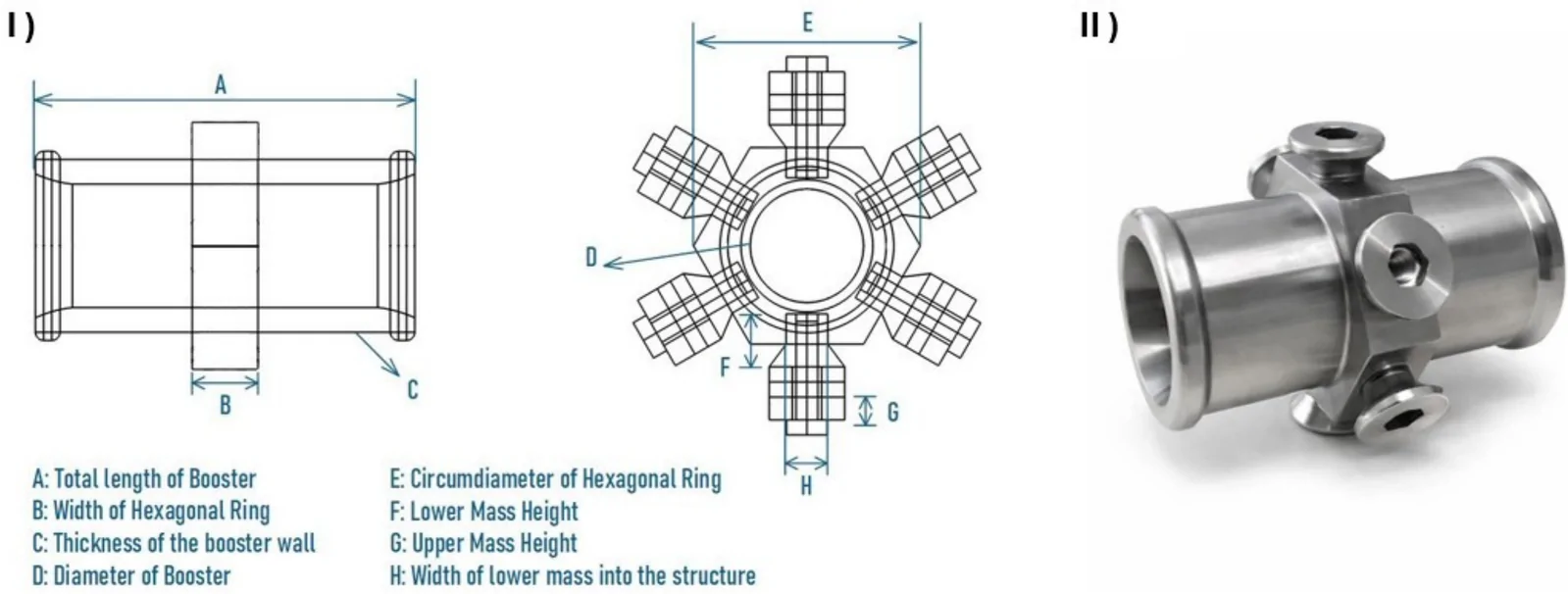
I) Schematic representation of the design parameters considered in booster optimization; II) Photograph of the fabricated booster unit.

A mid-frequency resonant unit (booster) has been designed and optimized through a total of ten parameters to maximize the pressure field in the center under flow-through conditions (Fig. 1). The optimization accounted for the reduced speed of sound in the bubbly liquid, experimentally and numerically estimated at approximately 900 m/s [68]. These parameters were systematically analyzed to determine operating conditions that improve cavitation efficiency while limiting energy consumption. In parallel, vibrational modes and structural responses were tuned to promote effective excitation of the target resonance mode and minimize energy losses. Together, these considerations formed the foundation for numerical simulations and the subsequent optimization strategy.

In addition, a multi-hole Venturi nozzle was incorporated to introduce hydrodynamic cavitation alongside acoustic cavitation [66]. Previous studies have demonstrated that coupling these two mechanisms can significantly enhance the energy efficiency of sonication. A key advantage of flow-through sonication systems is their ability to effectively integrate hydrodynamic cavitation with acoustic cavitation, thus intensifying cavitation activity under continuous operation.

The Venturi-shaped nozzle induces an acceleration of the local flow, and a corresponding pressure drop within the restricted region. Under the present operating conditions, this pressure reduction approaches, but does not consistently reach, the threshold required for sustained hydrodynamic cavitation. Superimposed acoustic excitation introduces periodic pressure oscillations, and during rarefaction phases, the instantaneous pressure near the Venturi decreases further, effectively reducing the cavitation threshold. The synergistic interaction between the hydrodynamic pressure drop and the acoustic field therefore promotes cavitation inception and intensifies bubble activity in the restricted zone [66].

Two Venturi-shaped nozzles were positioned upstream of the acoustic cavitation zone at nλ/2 from the sonicator inlet. The position of each nozzle relative to the cavitation field was optimized and validated through numerical simulations.

The flow velocity through the nozzle was insufficient to generate fully developed hydrodynamic cavitation independently. However, by the acoustic excitation of the reactor volume, the pressure fluctuation created will reduce the cavitation number locally at the orifice and thereby enhancing the cavitation initiation. At the applied mass flow rate (0.85 or 15 L/min), the five-hole Venturi-shaped nozzle generates a flow velocity in the narrow section approximately 3.6 or 8.8 m/s. This results in a cavitation number that is not sufficiently low to independently induce fully developed transient cavitation. However, in combination with a pulsating acoustic field (> 100 kPa), cavitation activity is significantly enhanced, promoting the initiation and collapse of larger bubbles, as well as increased turbulence and mixing of PFAS solutions.

### 2.3 Experimental setup

In this study, different reactor configurations and excitation frequencies have been evaluated. The batch reactor consisted of a beaker placed inside an aluminum chamber, where a 31 kHz horn sonotrode was immersed in the solution while a 21 kHz conical sonotrode was mounted in the center of the bottom plate of the chamber, thereby introducing ultrasonic irradiation from the top and bottom, respectively (dual frequency Batch system, DF-B). In addition, a booster unit operating at 135 kHz was designed and implemented to enable high-frequency flow-through sonication (SF-FT). The single high frequency flow through system was evaluated separately and further integrated into the dual frequency batch system for transition from batch to flow through system, resulting in a triple frequency hybrid system (TF-H).

**Fig. 2:**
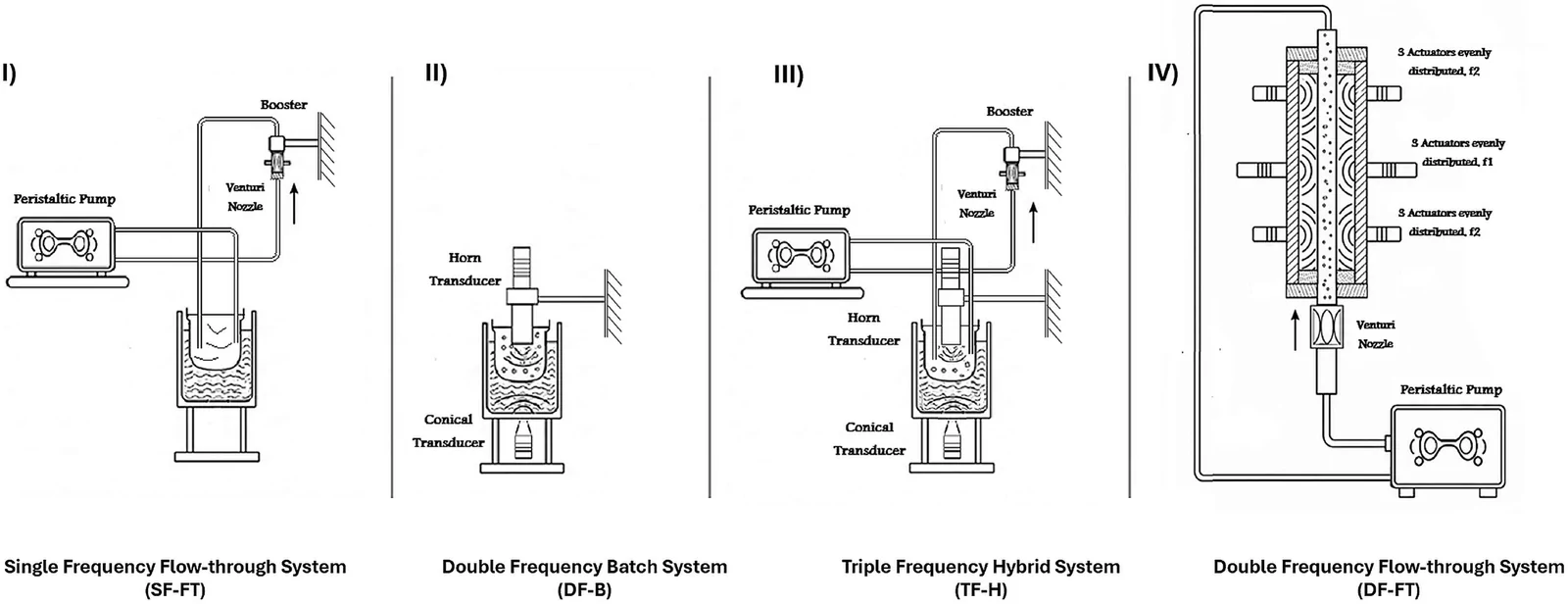
Different configurations of sonicators: I) Single-frequency flow-through sonicator (SF-FT), II) Dual-frequency Batch (DF-B), III) Triple-freq. Hybrid (TF-H): Dual frequency batch system + Single-frequency flow-through system, IV) Dual-frequency flowthrough system (DF-FT).

To investigate scalability and energy efficiency, a dual-frequency flowthrough configuration (DF-FT) was developed. The proposed sonicator combines hydrodynamic and acoustic cavitation. In this case, the Venturi-shaped nozzle [72] is located prior to the cavitation zone inside a stainless-steel tube equipped with three 21 kHz transducers positioned centrally and six 38 kHz transducers arranged in two additional rows evenly distributed around the circumference. The treatment capacity can be increased by connecting multiple flow-through sonicators in parallel to increase throughput or in series to increase cumulative cavitation exposure, thereby providing a modular pathway for process scale-up. Fig. 2 illustrates the schematic representations of the investigated reactor configurations.

The excitation of the ultrasonics transducers (f2, f3) was provided by EA PSI 9750–04 2U Power Supply, including power control and monitoring. The ultrasonics signal to each actuator was controlled by 600 V CoolGaN™ half-bridge evaluation boards triggered by signal generator square wave (05 V) for each frequency. Power to the conical transducer was supplied by a power amplifier (Yamaha, 300 W, 0–60 V) connected to a 1:10 transformer. The frequency and power were controlled using an Audiomatica CLIO 12 measurement system.

Table 1 presents the operating parameters of the investigated configurations, including excitation frequency and acoustic power density. All experiments were conducted with a total treatment duration of 2 h. However, the effective residence time within the active cavitation zone differed between the investigated flow-through configurations because only a fraction of the circulating liquid volume was directly exposed to intense cavitation conditions. The recirculation flow rate in the SF-FT system was maintained at 0.85 Lmin^−1^, while in the DF-FT system it was maintained at 15 Lmin^−1^. The SF-FT, DF-B, and TF-H systems were preheated, and experiments were initiated after the temperature stabilized between 32 and 35^◦^C. For the DF-FT experiments, the recirculation tank was placed in an ice bath, resulting in an initial temperature of approximately 21^◦^C.

**Table 1:** Operating conditions of the sonication systems investigated.

Parameter Unit		SF-FT	DF-B	TF-H	DF-FT
*f*_1_	[kHz]	–	(22.1, 21.3)	(22.1, 21.3)	23.8
*f*_2_	[kHz]	–	31.7	31.7	(38.5–––37.7)
*f*_3_	[kHz]	(135.4–––134.6)	–	(135.4–––134.6)	–
Power	[W]	25	80	105	175
Volume	[L]	0.26	0.20	0.26	3.5
Conc.	[mg/L]	1	1	1	10
*T*start	[^◦^C]	35	32	34	21
*T*end	[^◦^C]	51	43	51	41
*ΔT/Δt*	[^◦^C/min]	0.30	0.22	0.20	0.17

In the SF-FT configuration, the effective cavitation zone associated with the sonicator section accounted for approximately 3.0% of the total system volume (0.26 L), corresponding to an active cavitation volume of approximately 8 mL. Under the applied recirculation conditions, the total liquid volume passed through the cavitation zone approximately 392 times during the 2 h treatment period, resulting in an estimated cumulative cavitation exposure time of approximately 3.6 min.

In contrast, the effective cavitation volume in the DF-FT configuration represented approximately 10% of the total system volume (3.5 L), corresponding to an active cavitation volume of approximately 350 mL. Owing to the higher recirculation flow rate, the total liquid volume circulated through the cavitation region approximately 514 times during the treatment period, yielding an estimated cumulative cavitation exposure time of approximately 12 min.

In the DF-FT system, the flow-induced hydraulic power of 30 W was excluded from the reported acoustic power input, whereas the corresponding hydraulic contribution in the SF-FT configuration was negligible in comparison and estimated to be only on order of a few watts. In this setup, a higher concentration of PFAS of 10 mgL^−1^ was applied to investigate the effect of increased concentration to improve mass removal, when the system operated near the saturation regime. However, excessive formation of big bubbles adversely affected flow stability and cavitation uniformity under recirculating conditions. Therefore, an open mixing tank was implemented to minimize foam accumulation during recirculation, which was associated with the surface-active properties of PFAS.

### 2.4 PFAS analysis

PFAS degradation was evaluated by analyzing PFAS concentrations in untreated and treated solutions using liquid chromatography–tandem mass spectrometry (LC-MS/MS). The UPLC-MS/MS used was the Waters Acquity Ultra-Performance Liquid chromatograph coupled to a triple quadrupole mass spectrometer XEVO-TQS (Waters Corp., Milford, MA) equipped with a 100 mm × 2.1 mm C18 BEH column (1.7 µm particle size). A gradient mobile phase of (A) 2 mM ammonium acetate (95:5, methanol: ultrapure water) and (B) 2 mM ammonium acetate (MeOH) was used, the flow rate 0.30 mL min^−1^, and the injection volume 10 µL. The LC operated with an injection volume of 10 µL and the elution gradient started with 100% A, which decreased to 80%, 55%,20% and 5% at 1, 6, 13, 17 min, respectively. At 18 min, the A was again increased to 100% until the run was finished. The elution with mobile phase 100% B was varied accordingly. The MS/MS system worked in the Unispray ionization mode, and PFAS was quantified by a multiple reaction monitoring mode. Unispray conditions were: 1.0 kV impact voltage, 600 ^◦^C desolvation temperature, 1100 L/h desolvation gas flow, 150 L/h of cone gas flow, and 130 ^◦^C source temperature. Eleven PFAS compounds (PFBA, PFPeA, PFHxA, PFHpA, PFOA, PFNA, PFDA, PFBS, PFHxS, PFOS, and 6:2 FTS) were quantified. The details regarding nomenclature, list of abbreviations, and ionization and MS/MS mode for the analyzed PFAS are provided in the supplementary material (tables S1, and S2).

The limit of quantification corresponded to the lowest calibration standard, 5 ng/L, with a signal-to-noise ratio greater than 10. PFNA and 6:2 FTS were included because they formed part of the routine analytical target list, rather than because they were expected transformation products of PFOA or PFOS. Ultra-short-chain PFAS, including trifluoroacetic acid and perfluoropropanoic acid, were not included in the analytical method and were therefore not quantified.

### 2.5 Performance metrics

The removal efficiency of PFAS was first quantified by comparing concentrations of PFOX before and after treatment. The percentage removal was calculated as

%PFAS Removal = [PFAS]*i* − [PFAS]*t* × 100 (1).

[PFAS]*_i_*

where [PFAS]*_i_* and [PFAS]*_t_* represent the initial and final concentrations, respectively.

Although fractional removal provides a direct measure of degradation extent, it does not account for differences in energy input between operating conditions. Therefore, two complementary energy performance metrics were employed: energy yield (*Y*, mg/kWh) and input energy density (IED, kWh/L).

The energy yield was calculated as

*m*_removed_

*Y* =*Pt* (2).

where *m*_removed_ (mg) is the total mass of degraded PFAS, *P* (kW) is the electrical input power, and *t* (h) is the treatment time. This parameter represents the mass of PFAS removed per unit of electrical energy consumed and therefore provides a practical measure of process productivity and energy efficiency.

To enable comparison between systems operating with different volumes, the input energy density was additionally determined as

(3) $$\mathrm{IED}=\frac{\mathrm{Pt}}{V}$$

where *V* (L) is the total treated liquid volume. IED normalizes energy consumption with respect to treatment volume, allowing more rigorous comparison among reactor configurations operating at different power densities and scales. Lower IED values indicate more energy-efficient treatment.

In the flow-through configurations (SF-FT and DF-FT), the effective cavitation volume was smaller than the total circulating volume. Nevertheless, the total treated volume was used in the IED calculations to maintain consistency among the evaluated systems. The cavitation zone volume has been considered in power density calculation. The implications of this assumption are further discussed in the Discussion section.

For the DF-FT experiments, direct comparison with the other systems should be interpreted cautiously because the initial PFAS concentration differed significantly, resulting in different degradation kinetics. Consequently, the DF-FT results are discussed separately, primarily to evaluate the scalability potential of the proposed sonication approach.

## 3 Results and discussion

The results presented in this section correspond to optimized operating conditions that provided stable reactor performance and reproducible PFAS degradation outcomes.

The four reported configurations were selected following approximately 25 preliminary experiments conducted during the systematic development and optimization of the sonication systems. The screening process included different reactor configurations (e.g. single-frequency horn 21 kHz, and multiple-frequency flow-through system (20, 40, 210 kHz)), chemical additives (persulfate), temperature-control strategies (e.g. ice bath), and flowthrough operating conditions (e.g. against and towards gravity).

Repeated experiments and repeated sampling were performed across the different reactor configurations under stable operating conditions. Based on the average variability observed among these repeated measurements, a standard error of approximately ± 5% was considered a representative estimate for the reported PFOA and PFOS removal values. This standard error reflects the overall experimental and analytical variability observed across the dataset and should not be interpreted as the standard deviation of an equal number of independent replicates for each individual configuration.

### 3.1 Simulation validation via impedance analysis

**Fig. 3:**
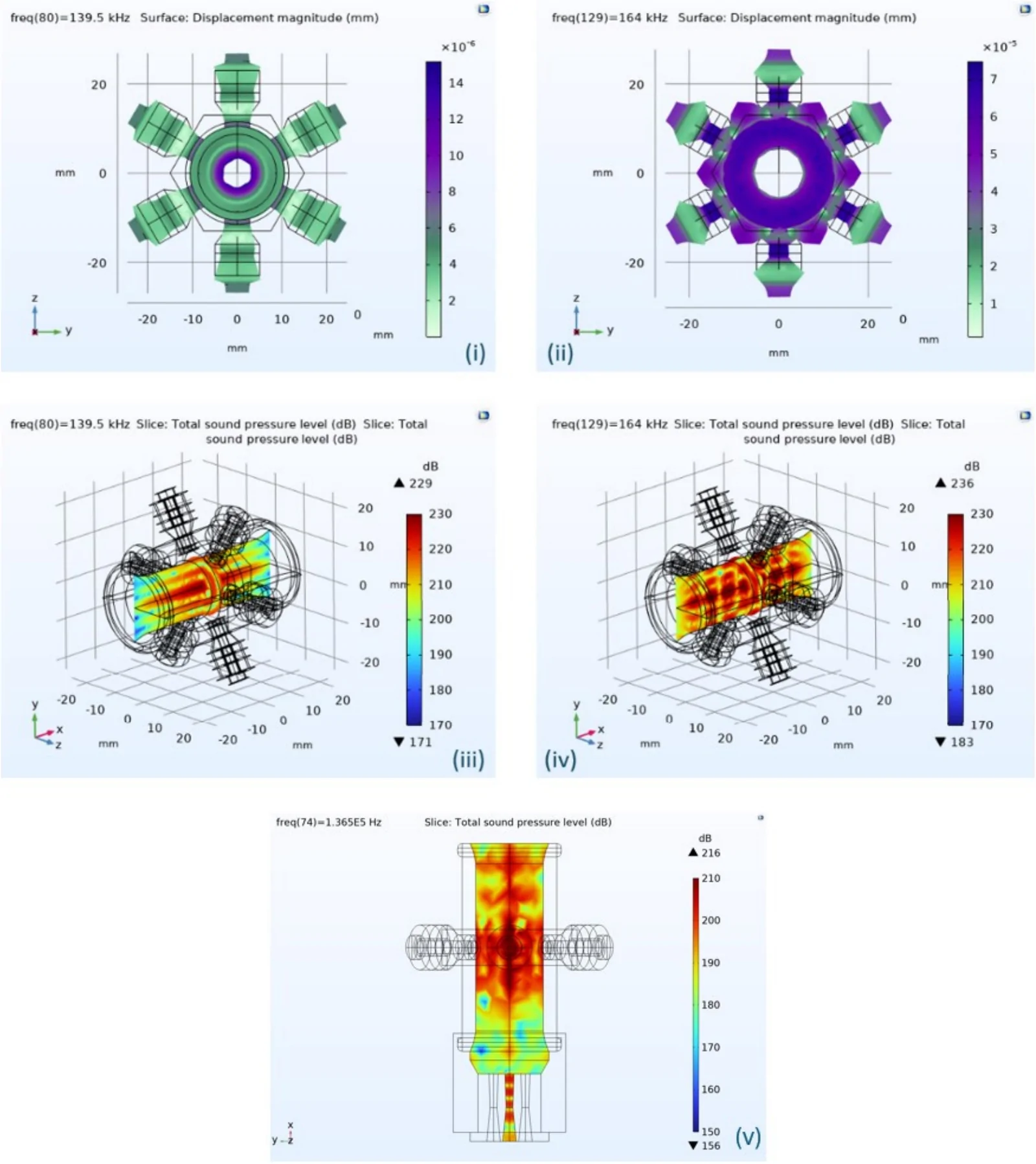
Modal vibrations (i, ii) and pressure distributions (iii, iv) of selected modes at 139.5 and 164 kHz, (v) The pressure fluctuation inside the Venturi at the vicinity of booster resonance frequency.

Fig. 3 i-iv, present two identified resonance frequencies, showing centrally localized pressure distributions and the corresponding vibrational mode shapes of the solid structure. The simulated frequencies of 139.5 and 164 kHz correspond to approximately 135 and 175 kHz, respectively, as observed in the impedance measurement spectra. Furthermore, Fig. 3v illustrates the pressure fluctuations within the Venturi region in the vicinity of the booster resonance frequency. Experiments conducted using only the Venturi integrated into the circulation pathway, without ultrasound irradiation, showed no measurable PFAS degradation. However, the incorporation of the Venturi into the ultrasonic flow-through system produced promising enhancement effects [66]. The simulation results suggest that the pressure fluctuations generated at the Venturi outlet are influenced by the pressure conditions within the sonication zone, thereby potentially enhancing cavitation activity and degradation performance.

**Fig. 4:**
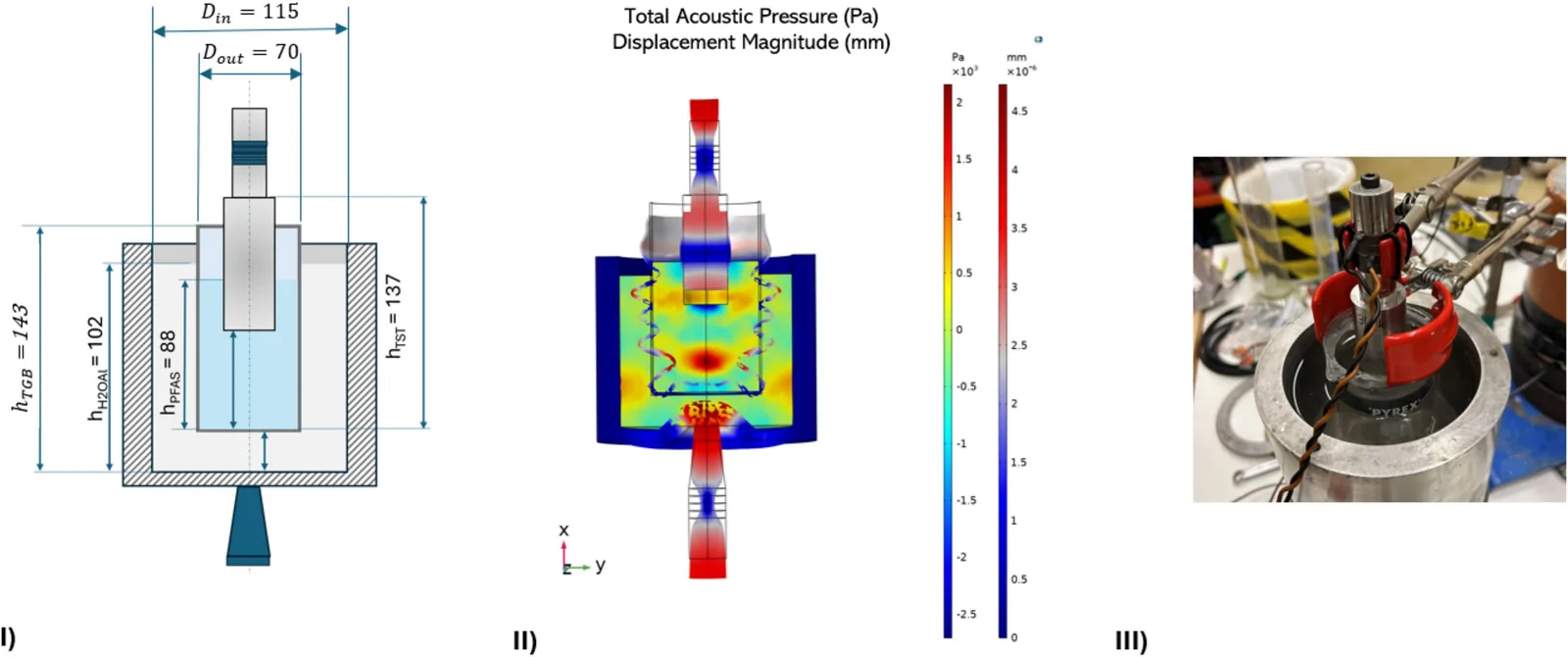
Dual-frequency batch sonication system: (I) schematic of the reactor geometry and principal dimensions, (II) simulated total acoustic pressure and structural displacement fields, and (III) photograph of the experimental setup.

Fig. 4 illustrates the dual-frequency batch sonication system, including the reactor geometry and dimensions, the simulated acoustic pressure and structural displacement fields, and the corresponding experimental setup.

### 3.2 Electrical impedance spectroscopy of the booster

Previous work by the authors [68] demonstrated that the minima in electrical impedance, close to the associated phase shift to zero, can be used as an initial estimation criterion to identify frequencies associated with efficient sonochemical activity. Consequently, the same methodology was applied in the present study as a first step to identify candidate operating frequencies of the ultrasonic booster.

**Fig. 5:**
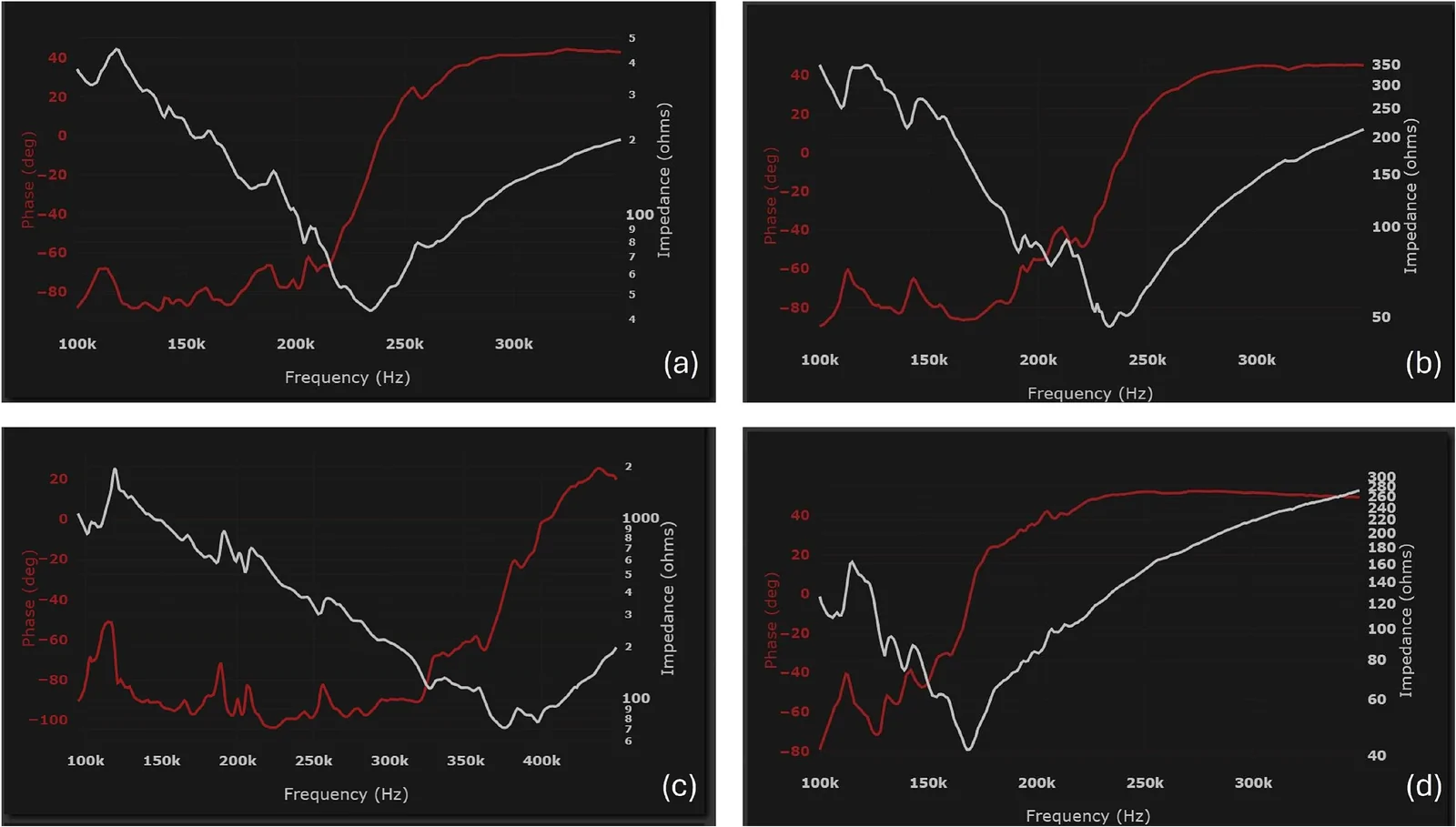
Measured electrical impedances of booster in case of: a, b)set of 3 sonotrodes parallel connected together, c) one sonotrode d)six sonotrodes connectd parallel together.

Fig. 5 presents the measured electrical impedance magnitude and phase of the booster for different transducer configurations: (a,b) three sonotrodes connected in parallel, (c) a single sonotrode, and (d) six sonotrodes connected in parallel. All configurations exhibit multiple impedance minima and associated phase transitions, indicating resonance regions of the coupled piezoelectric transducer–booster system. Increasing the number of parallel connected sonotrodes reduces the overall impedance and broadens the resonance region because of increased electrical loading and modified mechanical boundary conditions, whereas the single-sonotrode configuration shows higher impedance and sharper resonances.

Because a minimum electrical impedance does not necessarily correspond to maximum acoustic energy transfer into the liquid, several local minima were further evaluated using simultaneous sound-pressure measurements and aluminum-foil erosion tests. The global impedance minimum occurred near 175 kHz, while a local minimum was observed near 135 kHz. These frequencies were broadly consistent with the simulated high-intensity regions at 139.5 and 164 kHz. However, the measured pressure field and erosion results showed substantially stronger cavitation in the flow-through zone near 135 kHz.

The higher cavitation activity at 135 kHz indicates more effective fluid-structural coupling and acoustic energy transfer to the liquid. In contrast, the low electrical impedance but lower acoustic pressure at 175 kHz suggests predominantly reactive coupling, with energy stored in the structure and actuators rather than radiated efficiently into the fluid, resulting in actuator overheating. Therefore, 135 kHz was selected as the operating frequency because it provided the strongest and most reproducible cavitation conditions for the PFAS degradation experiments.

### 3.3 Effect of cavitation regime on PFAS degradation

The degradation and energy-normalized performance of the investigated systems after 2 h of treatment are summarized in Table 2. The degradation values varied among the investigated cavitation regimes, demonstrating that PFAS sonication depends not only on acoustic power or frequency magnitude, but also on cavitation intensity, spatial uniformity, and nonlinear multi-frequency interactions [19], [25], [33].

**Table 2:** Comparative degradation and energy-normalized performance.

System	PFOX	Removal^a^	Yield	teff	IED^b^		PD^c^					P_cal_^d^			η_cav_^e^	
		[%]	[mg/kWh]	[min]	[kWh/L]		[W/L]					[W]			[%]	
SF-FT	PFOA	55 ± 5	2.7 ± 0*.*14	3.6	0.19		3125			21±2			80±7			
	PFOS	66 ± 5	3.7 ± 0*.*19													
DF-B	PFOA	45 ± 5	0.5 ± 0*.*03	120	0.80		400			14±4			17±5			
	PFOS	40 ± 5	0.4 ± 0*.*02													
TF-H	PFOA	81 ± 5	1 ± 0*.*05	96	0.81		504			34±5			32±4			
	PFOS	77 ± 5	0.7 ± 0*.*04													
DF-FT	PFOA	36 ± 5	36 ± 1*.*8	12		0.10		500			95±10			46±3		
	PFOS	74 ± 5	62 ± 3*.*1													

a The reported uncertainties represent a dataset-wide estimate of experimental and analytical variability, based on the average deviation observed in repeated measurements across the investigated configurations.

b IED values are calculated based on the total treatment time of 2 h.

c PD values are defined as the averaged electrical input power per cavitation zone volume.

d Extended calorimetric estimate accounting for system and environmental losses.

e Cavitation electrical-to-process power-transfer efficiencies.

The single-frequency flow-through system (SF-FT) achieved 55% PFOA and 66% PFOS removal at a lower IED of 0*.*19 kWh*/*L. Although its fractional removal was lower than that of TF-H (81% for PFOA and 77% for PFOS), the effective cavitation exposure was only 3.6 min (96 min in TF-H), indicating efficient acoustic focusing and energy transfer within the optimized 135 kHz booster. In contrast, DF-B achieved only 45% PFOA and 40% PFOS removal at an IED of 0*.*80 kWh*/*L, despite continuous exposure throughout the treatment period. This lower performance suggests that prolonged low-frequency treatment alone was insufficient in the absence of effective interfacial renewal and a spatially uniform cavitation field [19], [36], [46]. The triple-frequency hybrid system (TF-H) achieved the highest fractional removal at an IED of 0*.*81 kWh*/*L. Its superior performance compared with the dual-frequency batch system (DF-B), despite their similar IED values, indicates that the improvement did not result solely from greater energy input. Instead, the low-frequency batch section promoted bulk mixing, bubble redistribution, and repeated cavitation exposure, while the 135 kHz flow-through booster provided a localized high-frequency region with a larger active interfacial area. Recirculation between these complementary cavitation environments, together with possible nonlinear interactions among the applied frequencies, likely enhanced interfacial renewal and PFAS transport to active cavitation zones [25], [38], [39].

The dual-frequency flow-through system (DF-FT), combining acoustic excitation at 21 and 38 kHz with a Venturi-induced hydrodynamic pressure reduction, achieved 74% PFOS and 36% PFOA removal at the IED, 0*.*10 kWh*/*L. The combined hydrodynamic and acoustic effects likely enhanced bubble nucleation, mixing, and continuous renewal of the bubble– liquid interface [50], [66], [72], particularly favoring PFOS because of its stronger surface activity and greater interfacial affinity [30]. However, DF-FT was operated at an initial PFAS concentration of 10 mg*/*L and a treated volume of 3*.*5 L, whereas the other systems were evaluated at 1 mg*/*L and considerably smaller volumes. Its purpose was therefore not to provide a direct percentage-removal comparison with the other configurations, but to assess treatment capacity, scale-up potential, and energy utilization under higher PFAS loading. At the higher concentration, progressive occupation of the bubble interface may have shifted degradation toward interfacial saturation and mass-transfer-limited behavior [32], [33], [34], [73]. This effect may have been more pronounced for PFOA because its interfacial affinity is weaker than that of PFOS.

The relative degradation of PFOS and PFOA reflects the combined influence of molecular structure and reactor-specific cavitation conditions. PFOS has greater surface activity and interfacial affinity than PFOA, favoring its accumulation at cavitation bubble interfaces. This behavior may explain the substantially higher PFOS removal in DF-FT, where Venturi-induced pressure reduction and low-frequency acoustic excitation continuously promoted bubble nucleation, collapse, and interfacial renewal [30], [32]. In TF-H, the combination of low-frequency batch cavitation and the 135 kHz flow-through booster provided complementary bubble populations and repeated exposure to distinct cavitation zones. The open batch surface may also have facilitated the release of larger bubbles, potentially favoring the transfer of smaller bubbles into the high-frequency booster; however, this interpretation requires direct validation of the bubble-size distribution.

The effective power transferred to the process was estimated calorimetrically by extending the conventional sensible-heating relation, *P_cal_ = mc_p_(dT/dt)*, to account for the complex and transient heat balance of the experimental systems. In addition to sensible heating of the process liquid, the analysis considered heat storage and transfer within the reactor structure and surrounding water jacket, losses through external circulation components and reservoirs, and heat exchange with the ambient environment or cooling system, as applicable. These contributions are inherently coupled and vary with temperature and operating conditions, introducing uncertainty into the calorimetric correction. Since the sonication systems were designed to concentrate the acoustic field within the active process volume, the major fraction of the acoustically transferred energy associated with cavitation was assumed to be dissipated within this region and subsequently redistributed through these thermal pathways. Moreover, simultaneous multifrequency excitation produced pronounced fluid agitation, which may substantially enhance convective heat transfer compared with non-sonicated conditions. The corrected calorimetric powers and corresponding electrical-to-process power-transfer efficiencies presented in Table 2 should therefore be regarded as approximate, and generally conservative, estimates.

**Fig. 6:**
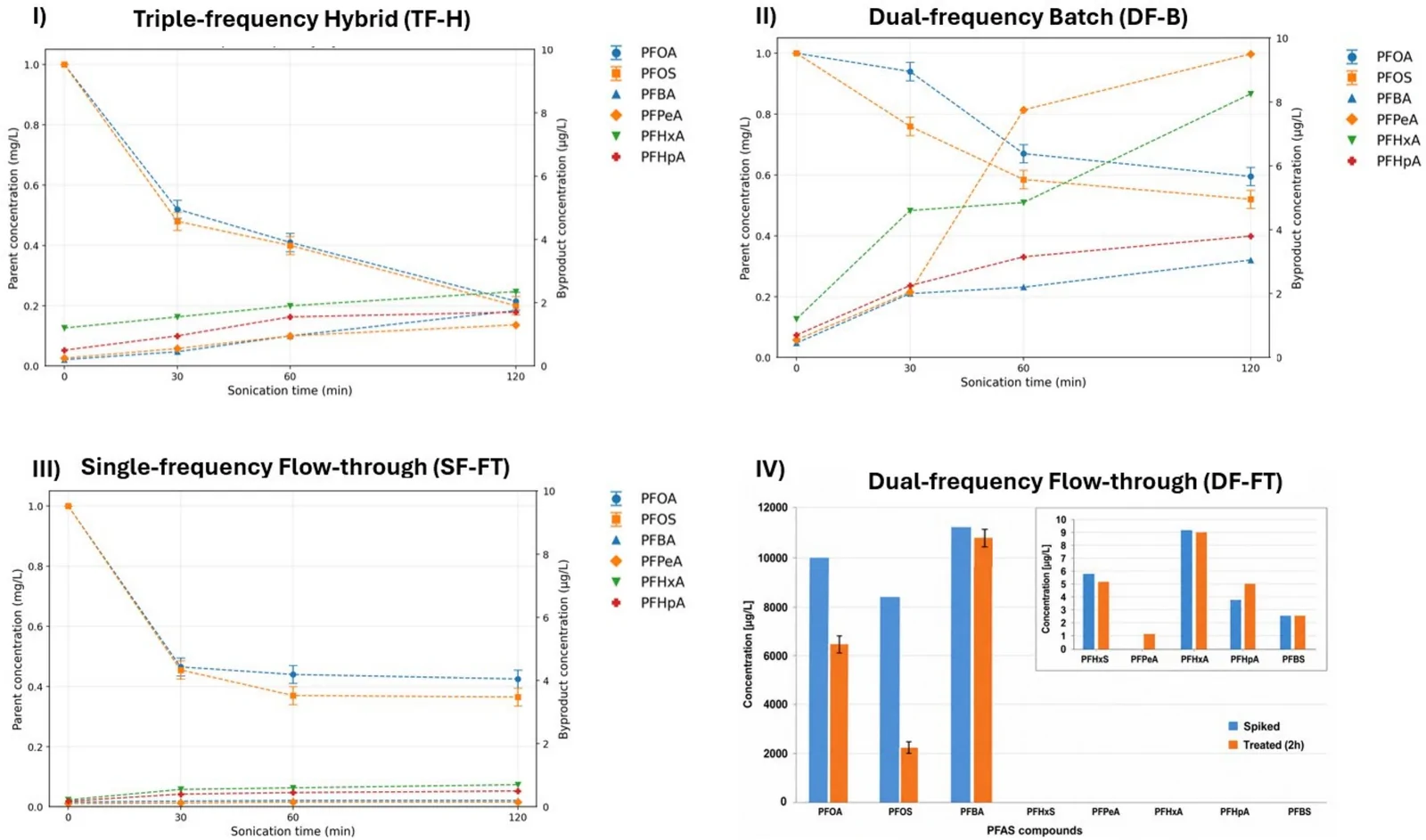
PFAS concentration versus exposure time for different sonication systems: (I) Triple-frequency mixed batch/flow-through (TF-H), (II) Dual-frequency batch (DFB), (III) Single-frequency booster flow-through (SF-FT), and (IV) Double-frequency flow-through (DF-FT). Parent PFAS concentrations are shown on the left axis, while transformation-product concentrations are shown on the right axis. Error bars for PFOA and PFOS represent a standard error of ± 5 percentage points in degradation, derived from repeated sampling and measurements under stable operating conditions.

The temporal concentration profiles in Fig. 6 support these performance differences. PFOS and PFOA concentrations decreased in all configurations, while PFHpA, PFHxA, PFPeA, and PFBA were formed during sonication (PFBA was also degraded in DF-FT). The formation of these C4–C7 compounds support a stepwise chain-scission pathway rather than direct and immediate mineralization.

The TF-H profiles showed the greatest overall reduction of both parent compounds, consistent with the complementary low- and high-frequency cavitation environments. SF-FT also produced substantial degradation despite its short effective exposure, confirming the effectiveness of the 135 kHz booster. By comparison, the lower removal and greater persistence of short chain products in DF-B suggest that initial chain shortening occurred without equally effective subsequent fragmentation.

The measured intermediates accounted for only a small fraction of the degraded parent compounds. In the SF-FT, DF-B, and TF-H systems, the detected C4–C7 products remained in the *µ*gL^−1^ range despite initial parent PFAS concentrations in the mgL^−1^ range. In the DF-FT system, their concentrations remained below 10 µgL^−1^ despite an initial concentration of 10 mgL^−1^. This limited accumulation suggests that the detected short-chain intermediates underwent further transformation into ultra-short-chain PFAS, fluoride ions, or other fluorinated products outside the analytical window.

Previous studies have shown that PFOA and PFOS degradation proceeds through pathways such as decarboxylation, desulfonation, and sequential chain shortening, which are commonly accompanied by fluoride release [31], [32]. It is therefore reasonable to infer that fluoride formation may also have occurred in the present systems. However, because fluoride release and total fluorine were not measured, the extent of defluorination and complete mineralization cannot be confirmed.

**Fig. 7:**
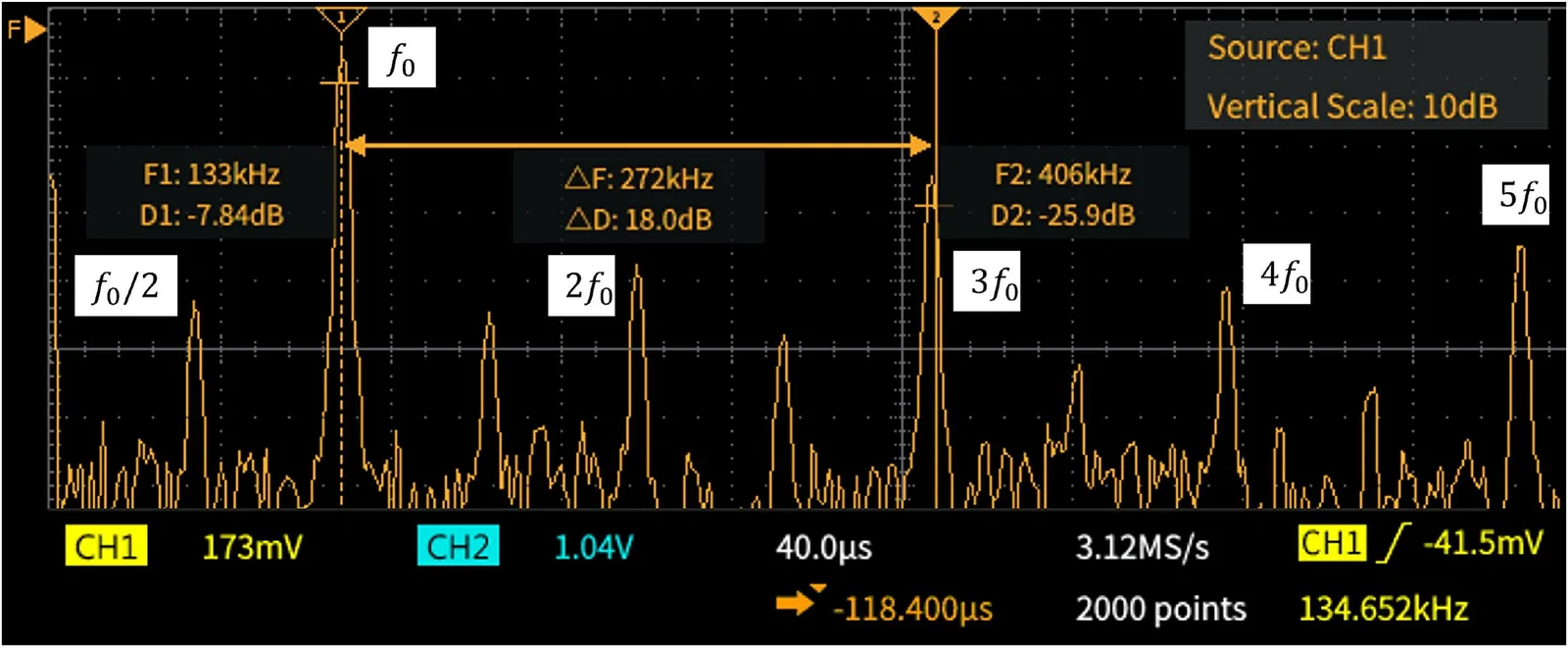
Pressure spectrum of booster measured by Tektronix TBS 1000C; indicating the harmonics of resonance frequency.

The favorable performance of SF-FT and TF-H can be further interpreted from the pressure spectrum of the 135 kHz booster shown in Fig. 7. In addition to the dominant fundamental frequency, pronounced odd harmonics and a distinct subharmonic component were observed, indicating nonlinear acoustic excitation and active bubble oscillation. Such spectral broadening can interact with a wider range of bubble sizes and resonance conditions, thereby increasing cavitation activity and sonochemical effectiveness [24], [36], [37]. The contribution of the booster therefore cannot be attributed solely to the additional electrical power; rather, it introduced a distinct high frequency, nonlinear cavitation regime that complemented the low-frequency fields.

**Fig. 8:**
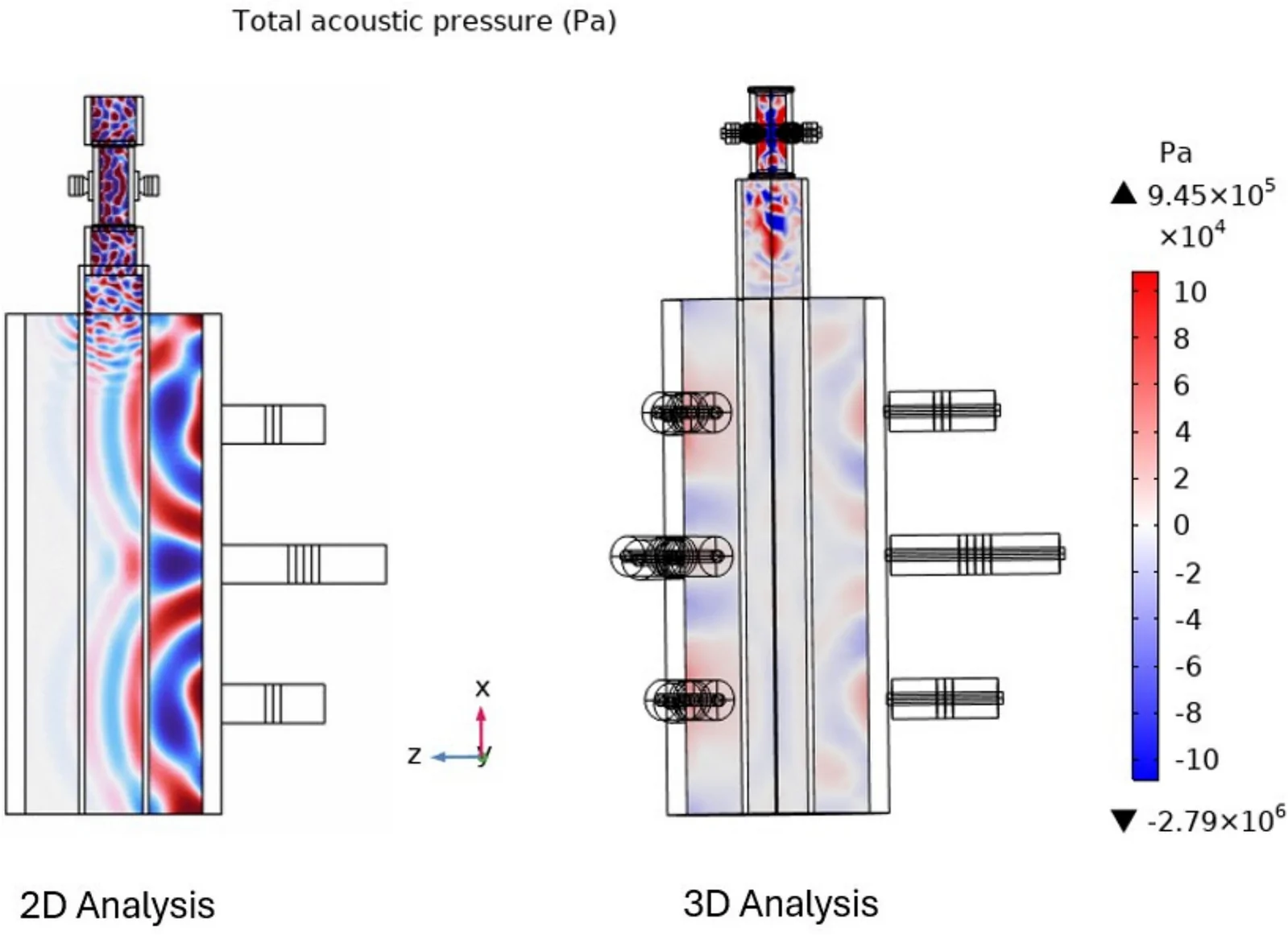
COMSOL Multiphysics simulation of the triple-frequency flow-through system, illustrating acoustic wave propagation and nonlinear wave interactions within the reactor.

Based on these findings, integration of the 135 kHz booster into the DF-FT system represents a logical development toward a triple-frequency flow-through configuration. As illustrated by the simulation in Fig. 8, this arrangement could combine the treatment capacity and favorable energy yield of the dual-frequency flow-through reactor with the pressure focusing, greater interfacial area, and nonlinear cavitation response of the high frequency booster.

However, operation of DF-FT at the higher PFAS concentration produced substantial formation and accumulation of large bubbles, requiring an open mixing tank to maintain stable recirculation. Such bubbles may cause acoustic shielding and reduce effective high-frequency energy transfer. Therefore, gas accumulation and bubble-size distribution must first be controlled before the booster can provide further cavitation enhancement. Future development should address gas separation, pressure control, recirculation design, and the relative positioning of the Venturi and booster. The configuration in Fig. 8 should therefore be regarded as a proposed scale-up strategy requiring further experimental validation.

## 4 Conclusion

This study systematically compared single-, dual-, and triple-frequency sonication systems operating under batch, flow-through, and mixed reactor configurations for PFAS degradation. The results demonstrate that sonochemical reactor performance is governed not only by the applied electrical power, but more fundamentally by the prevailing cavitation regime, acoustic field distribution, hydrodynamic conditions, and the efficiency of energy transfer into chemically active cavitation events.

Finite-element simulations enabled structural optimization of the booster unit (135 kHz), resulting in enhanced pressure amplification and improved acoustic focusing within the reactor domain. Electrical impedance characterization identified the effective operating frequency of the transducer–booster assembly, while spectral analysis confirmed the presence of a harmonic enriched acoustic field containing pronounced odd harmonics and subharmonic components, indicating nonlinear acoustic interactions and active cavitation behavior.

Among the investigated configurations, triple-frequency hybrid excitation achieved the highest fractional degradation efficiencies for both PFOS and PFOA, indicating enhanced cavitation intensity and synergistic acoustic coupling under multi-frequency operation. In contrast, the dual-frequency batch configuration promoted greater accumulation of shorter-chain intermediates, suggesting differences in degradation pathways and cavitation conditions between the investigated acoustic regimes. The relatively limited accumulation of intermediate products despite substantial long-chain PFAS degradation further suggests continued transformation of these intermediates rather than persistent accumulation within the reactor.

The dual-frequency flow-through (DF-FT) configuration combining hydrodynamic and acoustic cavitation demonstrated 74% PFOS mass removal together with the low input energy density (0.10 kWh/L). Considering that the applied concentration may lie near the reported kinetic transition region for PFAS sonolysis, the observed degradation performance is particularly significant because degradation behavior becomes increasingly influenced by interfacial saturation and mass-transfer limitations under these conditions.

## CRediT authorship contribution statement

**Sara Maghami:** Writing – original draft, Visualization, Validation, Software, Methodology, Investigation, Formal analysis, Data curation, Conceptualization. **Jean Noel Uwayezu:** Writing – review & editing, Resources, Methodology, Investigation, Conceptualization. **Ivan Carabante:** Resources, Funding acquisition, Conceptualization. **Örjan Johansson:** Writing – review & editing, Validation, Supervision, Resources, Project administration, Methodology, Investigation, Conceptualization.

## Declaration of competing interest

The authors declare that they have no known competing financial interests or personal relationships that could have appeared to influence the work reported in this paper.
